# Supplementary figures and images for: Nesprin-2 coordinates opposing microtubule motors during nuclear migration in neurons
Source: J Cell Biol. 2024 Aug 8;223(11):e202405032. doi: 10.1083/jcb.202405032 (PMC11310688; doi:10.1083/jcb.202405032)

**A**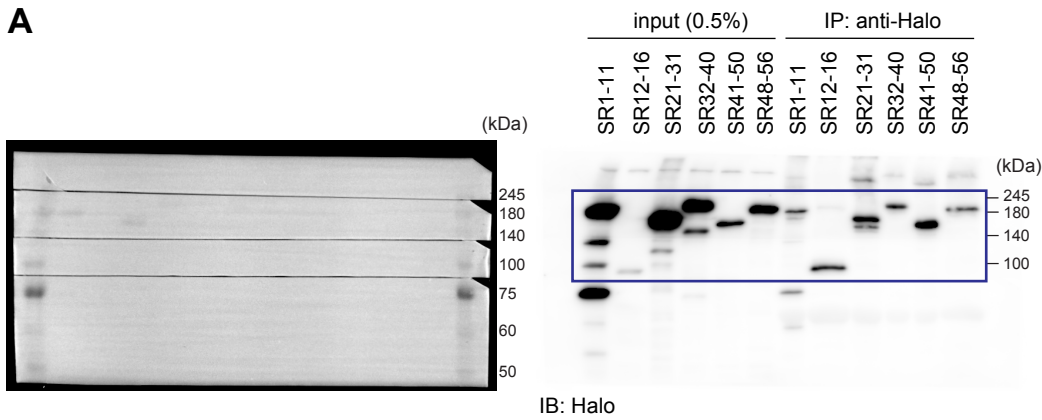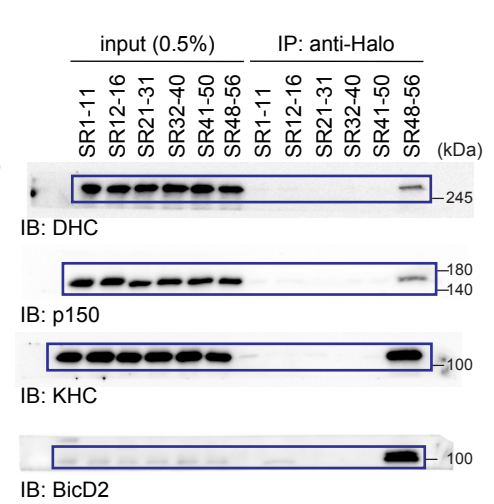**B**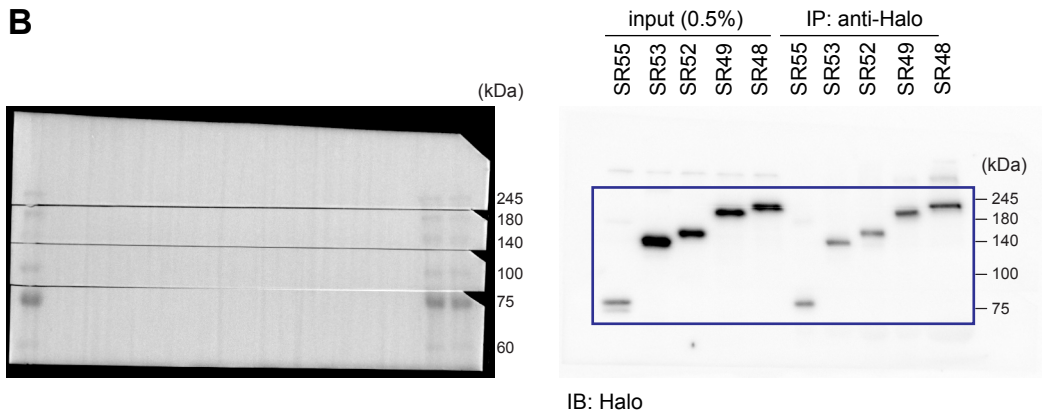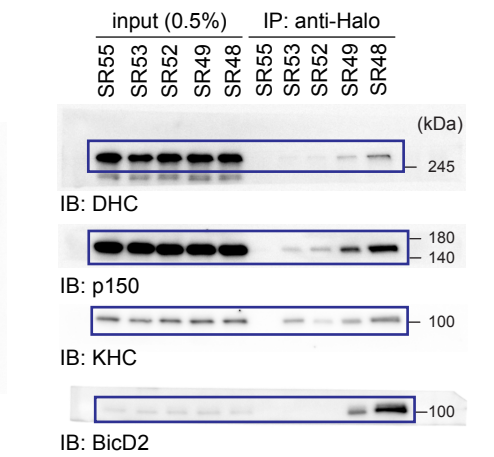**C**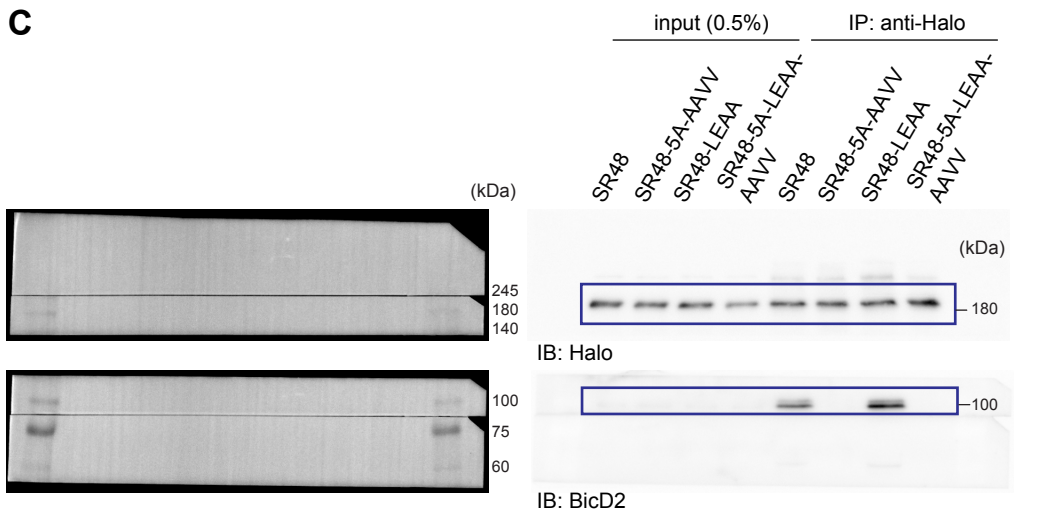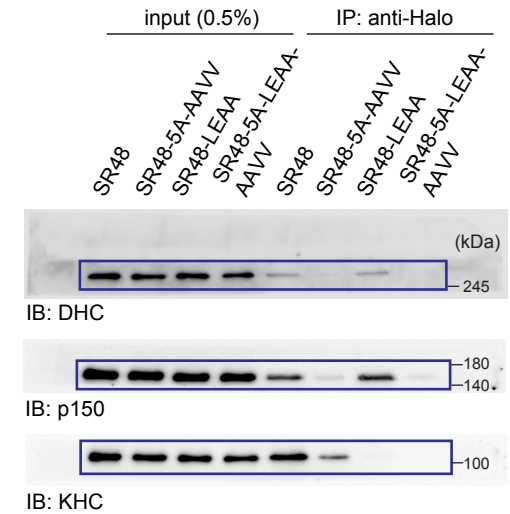**D**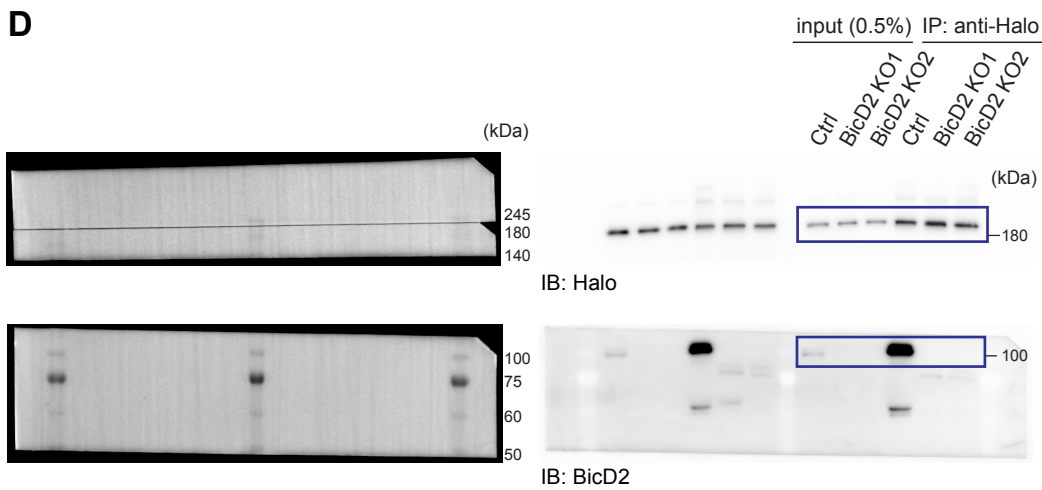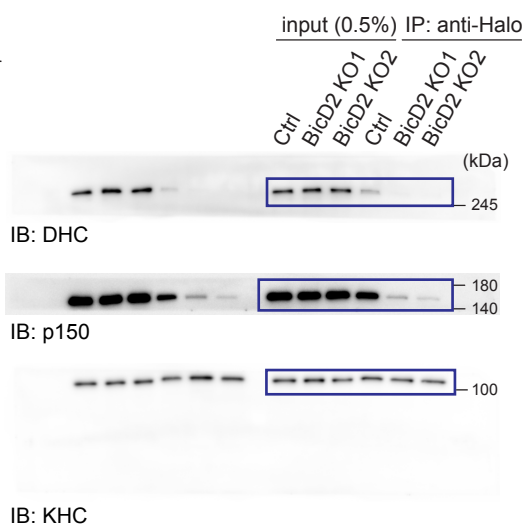

Supplement: SourceData F2 — is the source file for Fig. 2. [file JCB_202405032_SourceDataF2.pdf]

**A**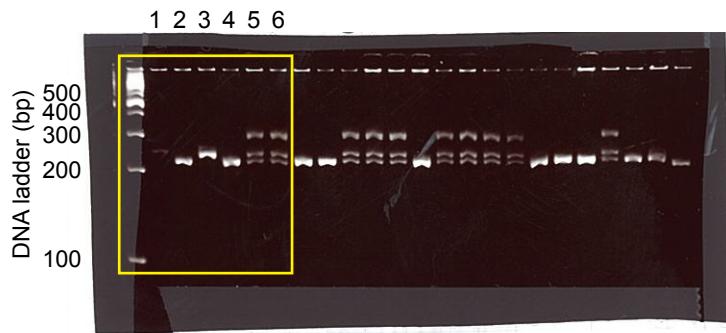**B**

IB: Nesp2

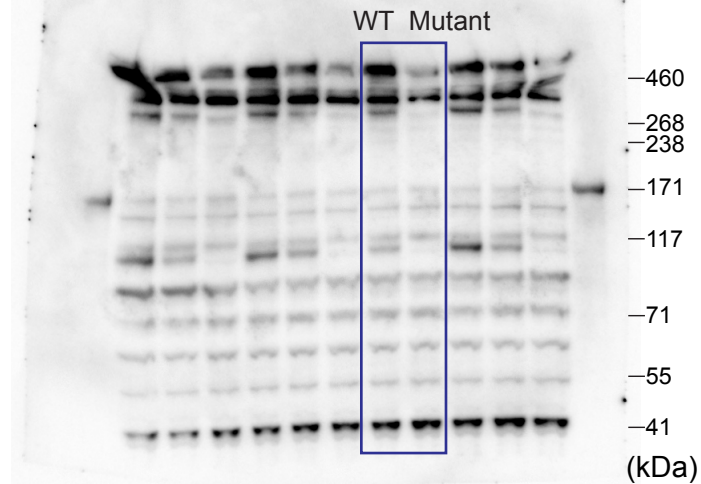IB:  $\beta$ -actin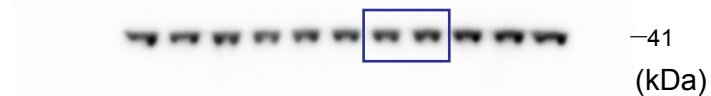

Supplement: SourceData FS2 — is the source file for Fig. S2. [file JCB_202405032_SourceDataFS2.pdf]
